# Physical, Mental, and Health Empowerment Disparities Across Chronic Obstructive Pulmonary Disease, Asthma, and Combined Groups and the Moderating Role of eHealth Literacy: Cross-Sectional Study

**DOI:** 10.2196/70822

**Published:** 2025-05-05

**Authors:** Jiaying Li, Xiaobing Wu, Yibo Wu, Daniel Yee Tak Fong, Yang Song, Siyi Xu, Changhwan Kim, Xiaohong Lin, Vinciya Pandian

**Affiliations:** 1 School of Nursing Johns Hopkins University Baltimore, MD United States; 2 Guangzhou Institute of Respiratory Health First Affiliated Hospital of Guangzhou Medical University Guangzhou China; 3 School of Public Health Peking University Beijing China; 4 School of Nursing University of Hong Kong Hong Kong China (Hong Kong); 5 School of Nursing Guangzhou University of Chinese Medicine Guangzhou China; 6 Ross and Carol Nese College of Nursing, The Pennsylvania State University; College of Medicine, The Pennsylvania State University; Hershey Center for Immersive Learning and Digital Innovation, The Pennsylvania State University University Park, PA United States

**Keywords:** chronic respiratory diseases, chronic obstructive pulmonary disease, asthma, health outcome, eHealth literacy

## Abstract

**Background:**

Nonpharmacological management plays a key role in enhancing the quality of life for individuals with chronic obstructive pulmonary disease (COPD), asthma, or both. However, disparities in their physical, mental, and health empowerment outcomes have not been fully explored, particularly in relation to the moderating effect of eHealth literacy.

**Objective:**

This study aims to assess these disparities and examine how eHealth literacy moderates them to guide the development of tailored nonpharmacological strategies.

**Methods:**

We analyzed data from 2 waves of the nationally representative “Psychology and Behavior Investigation of Chinese Residents” surveys to identify participants who self-reported asthma, COPD, or both. We assessed 5 physical outcomes (quality of life, physical activity, sleep quality, appetite, nicotine dependence), 4 mental outcomes (anxiety, depression, perceived stress, resilience), and 3 health empowerment measures (social support, self‐efficacy, eHealth literacy). Multiple regression with Holm-Bonferroni corrections revealed health disparities and the moderation effect of eHealth literacy.

**Results:**

This unfunded study enrolled 1044 participants between June 2022 and August 2023, with data analysis completed within 7 months following data collection. The sample included 254 (24.3%) participants with asthma, 696 (66.7%) participants with COPD, and 95 (9.1%) participants with both conditions. The mean age of the participants was 48.61 (SD 19.70) years, and 536 (51.3%) participants were male. Linear regression showed that individuals with both COPD and asthma had lower health-related quality of life and higher anxiety and depression compared with those with either condition alone (*b* ranges from –0.15 to 3.35). This group also showed higher nicotine dependence than asthma-only (*b*=0.88) and lower resilience than COPD-only groups (*b*=–0.76) (all adjusted *P*<.05). eHealth literacy significantly moderated the effect of the disease group on all outcomes except physical activity (all adjusted *P* for interaction <.05). Nine disease pairs showed disparities in both eHealth literacy groups, 4 only in high literacy, and 8 only in low literacy (all *P*<.05).

**Conclusions:**

Individuals with both COPD and asthma had poorer quality of life, greater anxiety and depression, higher nicotine dependence, and lower resilience, underscoring the need for integrated psychosocial and behavioral interventions. Although higher eHealth literacy was associated with improved quality of life, sleep quality, and resilience, it also widened disparities in anxiety and depression. Therefore, while enhancing eHealth literacy may help reduce overall health inequities among patients with chronic respiratory diseases, its potential adverse effects on mental well-being warrant careful attention. Moreover, lower eHealth literacy was linked to more pronounced disparities, indicating that outreach efforts and digital resources should be specifically designed to reach and empower vulnerable populations. Overall, our findings advocate for condition-specific, eHealth-enhanced care pathways that not only foster digital literacy but also integrate comprehensive mental health services, thereby mitigating health disparities among individuals with chronic respiratory diseases.

## Introduction

Chronic obstructive pulmonary disease (COPD) and asthma are major global health challenges. In 2019, COPD caused approximately 3.3 million deaths, while asthma affected around 262 million people [1,2]. In China, chronic respiratory diseases are the third leading cause of death among noncommunicable diseases, with an age-standardized prevalence rate of 4435 per 100,000 and a death rate of 76 per 100,000. Given its large population, these figures demonstrate a substantial disease burden [3]. Compared with other countries, China’s health care system—characterized by hospital-centered care and cultural attitudes emphasizing rapid treatment—can influence patients’ engagement with self-management, mental well-being, and other nonclinical outcomes related to COPD and asthma. Although both conditions are incurable, interventions that extend beyond disease-focused treatments—such as nonpharmacological strategies targeting quality of life, mental well-being, and patient empowerment—can enhance long-term outcomes, including reducing readmission rates and improving physical function [4-6]. However, because COPD and asthma progress differently, these approaches must be tailored to each condition’s distinctive physical, psychological, and self-management demands. Specifically, COPD progression, characterized by a continuous health decline, exacerbates symptoms and increases depression risk [7]. In contrast, asthma’s intermittent exacerbations disproportionately heighten anxiety [8]. For individuals with both COPD and asthma, the compounded challenges intensify physical and mental distress [9]. Recognizing these distinct disease profiles is essential to tailoring nonpharmacological strategies that foster long-term well-being and self-management.

However, existing research primarily emphasizes clinical outcomes disparities like mortality and hospitalization, overlooking comprehensive comparisons among COPD, asthma, and combined COPD-asthma, especially regarding psychosocial impacts. For instance, individuals with combined COPD-asthma report a lower quality of life compared with those with either condition alone [9]. Additionally, patients with COPD have higher in-hospital mortality rates compared with those with asthma [10]. Moreover, individuals with combined COPD-asthma experience more depression and fatigue than those with asthma alone, who are, in turn, more prone to anxiety [11,12]. Despite these insights, the literature often overlooks the broader effects of disease across physical, mental, and health empowerment domains, leading to generalized perspectives that may not reflect the unique disparities of each group. In China, factors such as a high prevalence of smoking, poor air quality, and a strong focus on hospital-based care may further exacerbate disease-specific differences in physical health, mental well-being, and patient empowerment. Our study addresses these gaps by incorporating a wider range of outcomes, including physical health, mental health, and health empowerment, to guide the development of more precise, person-centered nonpharmacological strategies.

eHealth literacy, defined as the ability to access and use digital health resources effectively, has emerged as a critical factor in empowering patients and improving their well-being [13]. Studies show that higher eHealth literacy improves the quality of life for patients with COPD but paradoxically worsens it for those with asthma [14,15], highlighting its complex and varied effects across respiratory diseases. Within China, the rapid expansion of internet and mobile technology adoption has fostered diverse eHealth initiatives [16]; yet, older adults—often with lower educational attainment—might have limited digital engagement. These gaps can influence how eHealth literacy differentially shapes disease outcomes. Additionally, research has not yet examined how eHealth literacy moderates the effects of disease conditions in the physical, mental, and empowerment domains. We assume that eHealth literacy moderates the relationship between disease type and patient outcomes, encompassing physical and behavioral, mental, and empowerment aspects. Different diseases demand varying levels of management complexity, with more challenging conditions often leading to poorer outcomes [9,10]. However, higher eHealth literacy can mitigate these challenges by equipping patients with robust self-management skills regardless of the specific chronic condition [17,18]. For example, a well-informed, eHealth-savvy patient with COPD may achieve nearly the same level of self-care empowerment as a patient with asthma, thereby narrowing potential gaps in physical health, mental well-being, and overall confidence and support. This study investigates these interactions to understand eHealth literacy’s influence on well-being, aiming to guide the design of tailored strategies that enhance engagement and health outcomes for more equitable and effective health care.

The aim of this study is to use pairwise comparisons to comprehensively examine health disparities among individuals with COPD, asthma, and combined COPD-asthma in physical and behavioral (health-related quality of life, physical activity, appetite, sleep, nicotine dependence), mental (anxiety, depression, stress, resilience), and empowerment (eHealth literacy, social support, self-efficacy) domains. Additionally, this study also aims to explore the moderating effects of eHealth literacy on these disparities, providing new insights into its role in addressing health inequalities. We hypothesize that these groups will differ in the measured outcomes and that eHealth literacy moderates disparities. These findings aim to identify each group’s specific needs, guiding targeted nonpharmacological strategies and literacy-based interventions to effectively address health disparities.

## Methods

### Study Design

This is a cross-sectional study. The study was reported in accordance with the CHERRIES (Checklist for Reporting Results of Internet E-Surveys) checklist [19].

### Setting

This study used data from 2 cross-sectional waves of the “Psychology and Behavior Investigation of Chinese Residents” studies [20,21]. Two nationwide web-based surveys were administered between June 20 and August 31 in both 2022 and 2023 across China. Stratified and quota sampling were used to ensure representation of diverse populations across regions and demographics, thereby enhancing generalizability [20]. The sample covered 148 cities in 2022 and 150 cities in 2023. Responses were considered invalid and excluded from the analysis if they were incomplete (eg, missing responses to critical survey items beyond a predefined threshold) or if they exhibited internal inconsistencies indicative of inattentiveness or nonsystematic responding. The survey did not determine unique visitors; calculate view, participation, or completion rates; use cookies; perform IP or log-file checks; require registration; or exclude submissions by atypical timestamps; incomplete surveys could not be submitted.

### Participants and Sample Size Calculation

Eligible participants were permanent Chinese residents who spent no more than 1 month per year abroad, were at least 18 years, and could understand and complete the questionnaire independently or with assistance. Exclusion criteria included confusion and psychiatric or cognitive impairments [20,21]. An additional inclusion criterion in this study was a diagnosis of either COPD or asthma.

The sample size was determined using the rule of thumb of at least 10 events per variable as recommended for valid statistical inferences [22], requiring a minimum of 90 subjects for each linear regression analysis with up to 9 variables. Additionally, because moderation analyses typically require about 4 times as many participants as main effects analyses [23], we required 360 subjects for each analysis of interaction effects. This study included 1044 participants, thereby fulfilling the sample size requirements.

### Variables and Measurements

#### Sociodemographics

Sociodemographic data were collected via structured questions with preset response options. Age was measured as a continuous variable (“How old are you?”). Gender was assessed with “What is your gender?” (options: Female, Male). Ethnicity was determined by asking “What is your ethnic background?” (options: Han, Ethnic minorities). Education was measured by “What is your highest level of educational attainment?” (options: No education, High school or below, Vocational/associate degree, Bachelor degree, Master degree or above). Monthly income was assessed with “What is your monthly income (in CNY)?” (options: ≤3000, 3001-6000, 6001-9000, ≥9001; an exchange rate of 1 CNY=US $0.14 is applicable). Occupational status was determined by “What is your current occupational status?” (options: Employed, Volunteer, Retired, Student, Unemployed). All variables were categorical except age, which was analyzed as a continuous variable.

#### Categorization of Disease Diagnoses

Participants self-reported their diagnoses of COPD or asthma through 2 questions in the national surveys. First, participants were asked, “Have you been diagnosed by a doctor with any of the following conditions?” For respondents who selected “Respiratory system diseases,” a follow-up question was presented: “What type of respiratory system disease do you have?” with options including COPD, asthma, chronic bronchitis, chronic pulmonary heart disease, and others. Participants who reported either COPD or chronic bronchitis were classified as having COPD. We classified self-reported chronic bronchitis as a COPD subtype in accordance with World Health Organization criteria [24]. Those who reported asthma were classified as having asthma, while participants who selected both (either COPD or chronic bronchitis) and asthma were categorized as having combined COPD and asthma.

#### Physical and Behavioral Well-Being

Physical well-being was evaluated using 5 measures: health-related quality of life, nicotine dependence, physical activity, appetite, and sleep quality.

Health-related quality of life was assessed with the EQ-5D-5L scale, which covers mobility, self-care, usual activities, pain/discomfort, and anxiety/depression. Each dimension is rated from no problems (1) to extreme problems (5), resulting in an index value ranging from −0.391 (worst) to 1.0 (full health) based on the Chinese 5L value set [25]. The Cronbach α value for the scale was 0.81 [26], and it was 0.84 in our sample.

Nicotine dependence was assessed using the 6-item Fagerström Test for Nicotine Dependence, which evaluates 6 aspects, including time to the first cigarette, handling smoking restrictions, intensity of smoking desires, daily cigarette quantity, increased morning smoking, and smoking despite illness. The scale scores ranged from 0 to 10, with higher scores indicating greater nicotine dependence. The Chinese version of 6-item Fagerström Test for Nicotine Dependence had a Cronbach α of 0.65 [27,28], whereas our sample showed a Cronbach α of 0.58.

Physical activity levels were assessed using the International Physical Activity Questionnaire-7. Basal metabolic time per week was calculated by summing the metabolic equivalents (METs) for activities of varying intensities: mild-intensity (3.3 METs), moderate-intensity (4.0 METs), and strenuous-intensity (8.0 METs). For each activity level, METs were multiplied by the average daily duration and the number of days per week, and total weekly MET minutes were calculated [29].

Appetite was assessed with the 4-item simplified nutritional appetite questionnaire, where each item is scored from 1 to 5, yielding total scores between 4 and 20: lower scores signify reduced appetite. The Chinese version of the simplified nutritional appetite questionnaire demonstrated satisfactory reliability and validity, with a Cronbach α of 0.69 [30]. In our sample, the Cronbach α was 0.75.

Sleep quality was evaluated using the Brief Pittsburgh Sleep Quality Index, which measures sleep efficiency, duration, disturbances, latency, and subjective quality. Scores range from 0 to 15, with higher scores reflecting poorer sleep quality. The Brief Pittsburgh Sleep Quality Index has demonstrated good reliability, with a Cronbach α of 0.77 reported in prior research [31]. In our sample, the Cronbach α was 0.63.

#### Mental Well-Being

Mental well-being was comprehensively assessed using validated scales to measure depression, anxiety, perceived stress, and resilience.

Depression was measured using the 9-item Patient Health Questionnaire (PHQ-9), a 9-item self-administered questionnaire. Each item is scored from 0 (not at all) to 3 (nearly every day), with total scores ranging from 0 to 27. Higher scores indicate more severe depressive symptoms. The Chinese PHQ-9 demonstrated excellent internal consistency (Cronbach α=0.86) [32], whereas our sample showed a Cronbach α of 0.92.

Anxiety levels were assessed with the 7-item General Anxiety Disorder scale, comprising 7 items that evaluate generalized anxiety symptoms over the past 2 weeks. Items are scored from 0 to 3, resulting in total scores between 0 and 21. Higher scores reflect greater anxiety severity. The Chinese 7-item General Anxiety Disorder showed excellent internal consistency (Cronbach α=0.89) [33], whereas our sample showed a Cronbach α of 0.93.

Perceived stress was evaluated using the 4-item Perceived Stress Scale, a 4-item instrument measuring stress perceptions over the past month. Each item is scored from 0 to 4, with total scores ranging from 0 to 16. Higher scores indicate higher stress levels. The Chinese 4-item Perceived Stress Scale demonstrated strong psychometric properties (Cronbach α=0.85) [34], whereas our sample showed a Cronbach α of 0.87.

Resilience was assessed with the Connor-Davidson Resilience Scale 2-item, which includes 2 items evaluating adaptability and recovery from hardship. Each item is rated on a 4-point scale, with total scores ranging from 0 to 8. Higher scores signify greater resilience. The Chinese Connor-Davidson Resilience Scale 2-item exhibited acceptable reliability (Cronbach α=0.63) [35], whereas our sample showed a Cronbach α of 0.73.

#### Health Empowerment

Health empowerment outcomes included eHealth literacy, perceived social support, and self-efficacy. eHealth literacy was assessed using the 5-item Chinese version of the eHealth Literacy Scale (eHEALS). Each item was rated on a Likert scale from 1 (strongly disagree), 3 (unsure), to 5 (strongly agree), resulting in total scores ranging from 5 to 25. Higher scores indicate greater eHealth literacy. The Chinese version of the eHEALS demonstrated excellent reliability, with a Cronbach α of 0.95, and our sample confirmed similar reliability with a Cronbach α of 0.94.

Perceived social support was measured using the perceived social support scale, a 3-item instrument rated on a 7-point Likert scale ranging from “extremely disagree” to “extremely agree.” Total scores ranged from 3 to 21, with higher scores indicating a greater level of perceived social support. This scale assessed support from family, friends, and others and demonstrated high internal consistency, with a Cronbach α of 0.89 [36]. In our sample, the Cronbach α was 0.86.

Self-efficacy was measured using the 3-item New General Self-Efficacy Short Form. Each item, scored from 1 (“very difficult”) to 5 (“strongly agree”), assesses confidence in achieving goals across different contexts. Higher scores indicate greater self-efficacy. The Chinese version demonstrated excellent psychometric properties (Cronbach α=0.94) [37], whereas our sample showed a Cronbach α of 0.89.

In summary, all assessed outcome variables (physical and behavioral health, mental well-being, and health empowerment), their assessment scales, and measurement properties are summarized in Multimedia Appendix 1.

### Data Collection

Data were collected in China from June 20 to August 31 in both 2022 and 2023. Trained surveyors, who received comprehensive training to ensure consistent questionnaire administration and ethical interactions with participants, administered the surveys. Quality control measures, including spot checks and real-time supervisory oversight, were implemented to maintain data integrity and accuracy. After collection, data underwent logical checks and systematic cleaning to verify consistency and enhance dataset reliability.

### Data Analysis

Data were consolidated into a database using Microsoft Excel (version 16.52) and subjected to quality control checks. Specifically, we verified data completeness and ensured that each response met a predefined threshold for key survey items, thereby including only valid and reliable data in the final analysis. Descriptive statistics summarized participants’ demographic profiles and health characteristics across COPD, asthma, and combined COPD-asthma groups. Normally distributed continuous variables were presented as means and SDs, while categorical variables were reported as frequency counts. To compare physical, mental, and health empowerment outcomes across the disease groups, linear regression analyses were conducted, adjusting for confounders including age, gender, ethnicity, education level, income, and occupation. Additionally, in the absence of established cut-off values for the short-form Chinese eHEALS, previous studies have suggested using median or midpoint splits [38]. Given the balanced Likert structure with equal intervals and symmetry and the potential overrepresentation of high eHealth literacy in our web-based sample, we adopted a midpoint split. Then, eHealth literacy scores (ranging from 5 to 25) were categorized into high (>15) and low (≤15) groups. This classification was used to examine the moderating effects of eHealth literacy on the relationships between disease groups and outcomes while controlling for the same set of confounders.

Given the multiple pair-wise comparisons across 3 disease groups and 12 outcomes, a total of 36 comparisons were performed. To control for the increased risk of type I errors (false positives) due to multiple tests, Holm-Bonferroni correction was applied to adjust the *P* values. This step ensures that the family-wise error rate is maintained at the desired significance level, thereby enhancing the reliability of the identified significant effects. Similarly, for the interaction effects between the disease group and eHealth literacy across the 12 outcomes, Holm-Bonferroni adjusted *P* values were also calculated to account for multiple interaction tests.

All statistical analyses, including descriptive statistics, regression models, and *P* value adjustments, were performed using R software (version 4.1.1; R Foundation for Statistical Computing). A 2-tailed significance level was set at *P*<.05 after Holm-Bonferroni correction to determine statistical significance.

### Ethical Considerations

The dataset was collected with approval from the Ethics Research Committee of the Health Culture Research Center of Shaanxi (approval numbers JKWH-2022-02 and 2022-K050) and from the Ethics Research Committee of Shandong Provincial Hospital (approval no. SWYX:2023-198). Participants provided informed consent, and anonymity and confidentiality were assured [20,21]. The present secondary analysis used fully deidentified data and was exempt from further review. Permission to access and analyze the dataset was obtained from the original data custodian under license.

## Results

### Respondents’ Characteristics

**Figure 1:**
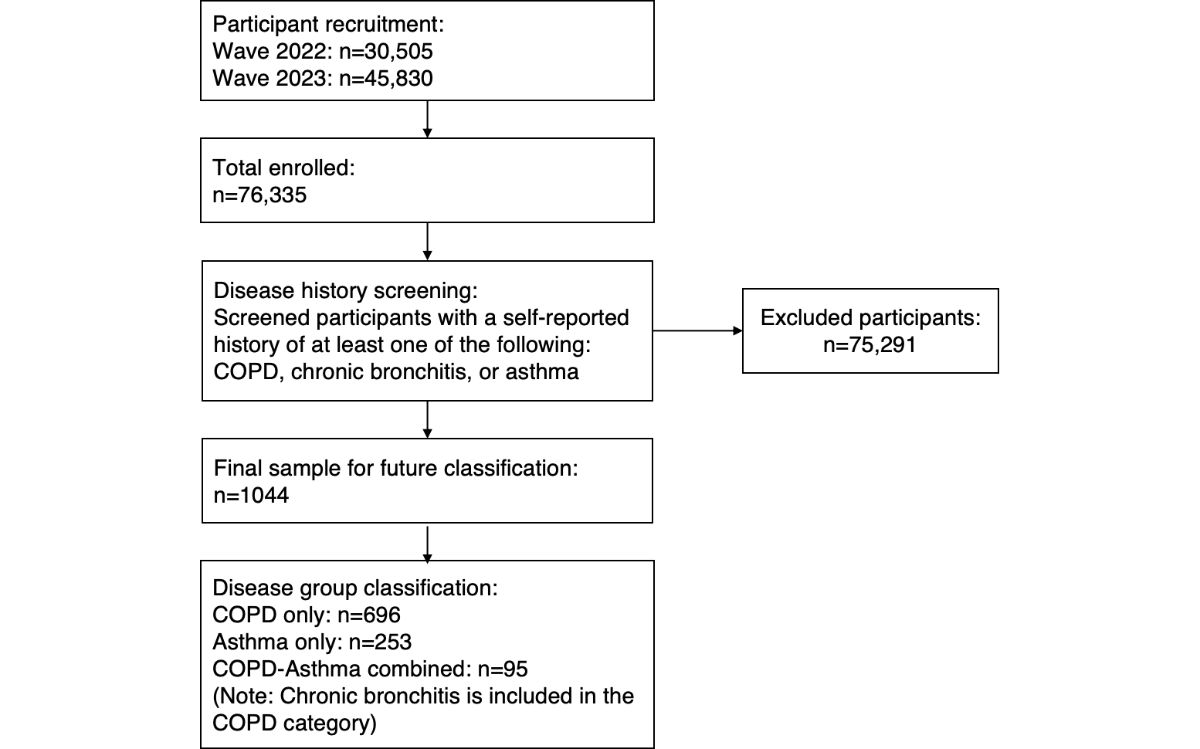
Flowchart of participant screening and disease classification. COPD: chronic obstructive pulmonary disease.

A total of 30,505 and 45,830 responses were collected in 2022 and 2023, respectively. Participants with a confirmed diagnosis of COPD or asthma were selected for analysis in our study. After rigorous screening, 1044 participants were included—244 (23.4%) participants from 2022 and 800 (76.6%) participants from 2023. Figure 1 illustrates the sample process used to derive the final analytical sample in this study.

The study included 1044 participants (mean age 48.6 years, SD 19.7), predominantly of Han ethnicity (n=910, 87.2%). The gender distribution was nearly equal (men: n=536, 51.3%; women: n=508, 48.7%). Participants were grouped into asthma (n=253, 24.2%), COPD (n=696, 66.7%), and combined COPD-asthma (n=95, 9.1%). Additional demographic details, including education, income, and occupation, are presented in Table 1.

**Table 1:** Participants’ demographics and disease group (n=1044).

Variable			Values
Age (years), mean (SD)			48.61 (19.7)
**Ethnicity, n (%)**			
	Ethnic minorities	134 (12.8)	
	Han	910 (87.2)	
**Gender, n (%)**			
	Women	508 (48.7)	
	Men	536 (51.3)	
**Highest level of education, n (%)**			
	No education	80 (7.7)	
	High school or below	403 (38.6)	
	Vocational or associate degree	281 (26.9)	
	Bachelor degree	233 (22.3)	
	Master degree or above	47 (4.50)	
**Monthly income (in CNY; 1 CNY=US $0.14), n (%)**			
	≤3000	330 (31.6)	
	3001-6000	412 (39.5)	
	6001-9000	147 (14.1)	
	≥9001	155 (14.8)	
**Occupational status, n (%)**			
	Employed	351 (33.6)	
	Volunteer	128 (12.3)	
	Retired	279 (26.7)	
	Student	159 (15.2)	
	Unemployed	127 (12.2)	
**Disease group, n (%)**			
	Asthma	253 (24.2)	
	COPD^a^	696 (66.7)	
	Combined COPD-asthma	95 (9.1)	

^a^COPD: chronic obstructive pulmonary disease.

### Health Disparities Across Disease Groups

Regression analysis identified no significant difference between COPD and asthma. However, several significant differences in physical and mental well-being outcomes were found between the combined COPD-asthma group and the groups with COPD alone or asthma alone, after adjusting for social determinant factors and applying the Holm-Bonferroni correction. A detailed comparison of outcomes across disease groups is provided in Table 2.

**Table 2:** Linear regressions of disease groups on physical and behavioral, mental, and health-empowerment outcomes (n=1044). Disease groups were taken into the regression model as independent variable, while the health outcomes were taken as dependent variable one by one. Each regression was adjusted by age, gender, ethnicity, highest level of education, monthly income, and occupational status.

Outcomes			COPD^a^ versus asthma									Combined COPD-asthma versus asthma									Combined COPD-asthma versus COPD						
			*b*		SE		*P* value		Adjusted *P* value		*b*			SE		*P* value		Adjusted *P* value		*b*			SE		*P* value		Adjusted *P* value
**Physical and behavioral well-being**																											
	Health-related quality of life	–0.03		0.02		.12		>.99		–0.15			0.03		<.001		<.001		–0.13			0.02		<.001		<.001	
	Physical activity	–368.3		268.19		.17		>.99		–1331.42			441.16		.003		.08		–963.12			394.66		.02		.36	
	Sleep quality	0.18		0.24		.47		>.99		0.5			0.4		.22		>.99		0.32			0.36		.37		>.99	
	Appetite	0.05		0.25		.83		>.99		–0.47			0.36		.19		>.99		–0.53			0.31		.09		>.99	
	Nicotine dependence	0.46		0.19		.02		.36		0.88			0.28		.001		.03		0.43			0.23		.07		>.99	
**Mental well-being**																											
	Depression	0.09		0.45		.84		>.99		3.35			0.74		<.001		<.001		3.26			0.66		<.001		<.001	
	Anxiety	0.06		0.37		.88		>.99		2.85			0.61		<.001		<.001		2.8			0.54		<.001		<.001	
	Perceived stress	0.24		0.24		.32		>.99		0.96			0.39		.02		.350		0.72			0.35		.04		.80	
	Resilience	0.05		0.16		.76		>.99		–0.71			0.24		.003		.084		–0.76			0.2		<.001		<.001	
**Health empowerment**																											
	eHealth literacy	–0.96		0.44		.03		.62		–0.48			0.64		.46		>.99		0.49			0.54		.37		>.99	
	Social support	–0.43		0.3		.16		>.99		–0.91			0.5		.07		>.99		–0.48			0.44		.28		>.99	
	Self-efficacy	–0.59		0.2		.003		.08		–0.61			0.32		.06		>.99		–0.02			0.29		.94		>.99	

^a^COPD: chronic obstructive pulmonary disease.

Specifically, participants in the combined COPD-asthma group, when compared with those in asthma and COPD groups, exhibited a lower health-related quality of life (vs asthma: *b*=–0.15, adjusted *P*<.001; vs COPD: *b*=–0.13, adjusted *P*<.001), and higher levels of anxiety (vs asthma: *b*=2.85, adjusted *P*<.001; vs COPD: *b*=2.80, adjusted *P*<.001), depression (vs asthma: *b*=3.35, adjusted *P*<.001; vs COPD: *b*=3.26, adjusted *P*<.001). Furthermore, nicotine dependence was significantly higher in the combined COPD-asthma group than in the asthma group (*b*=0.88, adjusted *P*=.03), while resilience was lower compared with the COPD group (*b*=–0.76, adjusted *P*<.001).

### Moderation Effect of eHealth Literacy on Health Disparities Across Disease Groups

**Figure 2:**
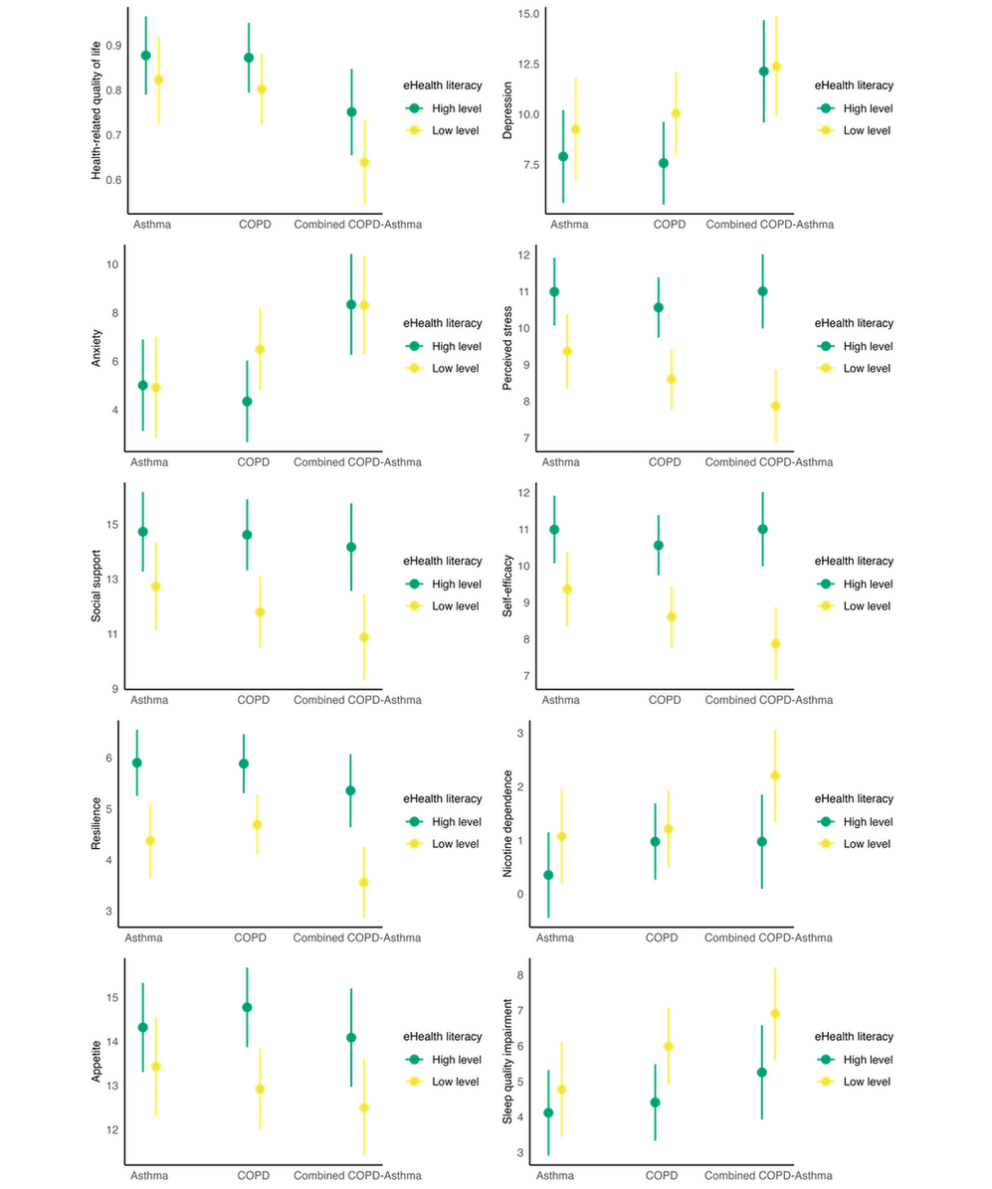
Visualization of eHealth literacy moderation on health disparities across disease groups. COPD: chronic obstructive pulmonary disease.

eHealth literacy levels significantly moderated the associations between disease groups and all outcomes (all adjusted interaction *P* values <.05), except for physical activity (interaction *P*=.14). These interaction effects are visualized in Figure 2.

In both high and low eHealth literacy groups, several disease pair comparisons showed significant differences in the same outcomes. The combined COPD-asthma group consistently exhibited lower health-related quality of life, higher levels of anxiety and depression, and poorer sleep quality compared with both the asthma and COPD groups (all *P*<.05). However, the health disparities in health-related quality of life and sleep quality were amplified in the low eHealth literacy group for the combined COPD-asthma group, as indicated by larger effect sizes (*b*), detailed in Table 3. Conversely, for anxiety and depression, the combined COPD-asthma group’s health disparities were narrowed in the low eHealth literacy group, with smaller effect sizes (*b*) observed. Additionally, the combined COPD-asthma group had lower resilience than the COPD group only across both high and low eHealth literacy groups, with these disparities being more pronounced in the low eHealth literacy group, as reflected by larger effect sizes (*b*).

**Table 3:** Moderation effects of eHealth literacy on disparities between disease group and outcomes.

Outcomes and disease groups							High eHealth literacy										Low eHealth literacy											*P* value for interaction					Adjusted *P* value for interaction
							*b*			SE			*P* value			*b*				SE			*P* value										
**Physical and behavioral well-being**																																	
	**Health-related quality of life**																										.001					.003	
				COPD^a^ versus asthma		0.00			0.02			.83			–0.02				0.04			.71											
				Combined COPD-asthma versus asthma		–0.12			0.03			<.001			–0.19				0.06			.002											
				Combined COPD-asthma versus COPD		–0.13			0.03			<.001			–0.17				0.05			<.001											
	**Physical activity**																										.137					.137	
				COPD versus asthma		–286.30			245.55			.24			–130.68				300.10			.66											
				Combined COPD-asthma versus asthma		–516.08			372.54			.17			–266.88				423.98			.53											
				Combined COPD-asthma versus COPD		–229.78			329.54			.49			–136.19				337.38			.69											
	**Sleep quality**																										<.001					<.001	
				COPD versus asthma		0.10			0.35			.77			1.16				0.49			.02											
				Combined COPD-asthma versus asthma		1.11			0.53			.04			2.24				0.69			.001											
				Combined COPD-asthma versus COPD		1.00			0.47			.03			1.08				0.55			.048											
	**Appetite**																										<.001					<.001	
				COPD versus asthma		0.61			0.26			.02			–0.49				0.45			.28											
				Combined COPD-asthma versus asthma		–0.09			0.40			.82			–1.15				0.64			.07											
				Combined COPD-asthma versus COPD		–0.70			0.35			.048			–0.66				0.51			.20											
	**Nicotine dependence**																										.007					.014	
				COPD versus asthma		0.62			0.23			.007			0.12				0.33			.71											
				Combined COPD-asthma versus asthma		0.73			0.35			.04			0.82				0.47			.08											
				Combined COPD-asthma versus COPD		0.11			0.31			.72			0.70				0.37			.06											
**Mental well-being**																																	
	**Depression**																										<.001					<.001	
			COPD versus asthma		–0.53			0.64			.41			0.97				0.93			.30												
			Combined COPD-asthma versus asthma		3.88			0.97			<.001			4.43				1.31			.001												
			Combined COPD-asthma versus COPD		4.41			0.86			<.001			3.46				1.04			.001												
	**Anxiety**																										<.001					<.001	
				COPD versus asthma		–0.88			0.53			.10			1.65				0.79			.04											
				Combined COPD-asthma versus asthma		3.05			0.80			<.001			4.19				1.11			<.001											
				Combined COPD-asthma versus COPD		3.93			0.71			<.001			2.54				0.89			.005											
	**Perceived stress**																										<.001					<.001	
				COPD versus asthma		–0.34			0.24			.15			–0.79				0.41			.05											
				Combined COPD-asthma versus asthma		–0.04			0.36			.92			–1.86				0.57			.001											
				Combined COPD-asthma versus COPD		0.31			0.32			.34			–1.07				0.46			.02											
	**Resilience**																										<.001					<.001	
				COPD versus asthma		0.08			0.17			.638			0.22				0.28			.45											
				Combined COPD-asthma versus asthma		–0.45			0.26			.085			–1.20				0.40			.003											
				Combined COPD-asthma versus COPD		–0.53			0.23			.022			–1.41				0.32			<.001											
**Health empowerment**																																	
	**Social support**																										<.001					<.001	
				COPD versus asthma		0.10			0.36			.784			–1.03				0.68			.13											
				The combined versus asthma		–0.42			0.54			.437			–2.13				0.95			.03											
				The combined versus COPD		–0.52			0.48			.279			–1.10				0.76			.15											
	**Self-efficacy**																										<.001					<.001	
				COPD versus asthma		–0.34			0.24			.152			–0.79				0.41			.05											
				The combined versus asthma		–0.04			0.36			.920			–1.86				0.57			.001											
				The combined versus COPD		0.31			0.32			.340			–1.07				0.46			.02											

^a^COPD: chronic obstructive pulmonary disease.

In the high eHealth literacy group exclusively, 4 disease pair comparisons reached significance. Both COPD and the combined COPD-asthma group had higher nicotine dependence than the asthma group, while COPD patients reported better appetite compared with those in asthma and combined COPD-asthma groups (all *P*<.05).

In the low eHealth literacy group, 8 disease pair comparisons were significant only within this subgroup. The combined COPD-asthma group had lower perceived stress and self-efficacy compared with both asthma and COPD groups, and reduced social support and resilience relative to the asthma group. Additionally, COPD patients had higher anxiety and worse sleep quality than those with asthma (all *P*<.05).

## Discussion

### Principal Findings

This study examines disparities in physical, mental, and empowerment health outcomes across COPD, asthma, and combined COPD-asthma groups, with a focus on eHealth literacy’s moderating effects. The combined COPD-asthma group experienced significantly poorer health-related quality of life, lower physical activity, higher levels of depression and anxiety, greater perceived stress, reduced resilience compared with the COPD and asthma groups, and higher nicotine dependence than asthma alone. Patients with COPD had lower eHealth literacy and self-efficacy but greater nicotine dependence than those with asthma. These findings highlight the need for tailored support programs addressing each patient group’s unique needs, especially the combined COPD-asthma group with consistently worse outcomes. Notably, eHealth literacy moderated health disparities: lower levels exacerbated disparities in physical health, sleep quality, and resilience but mitigated those in anxiety and depression. This suggests that enhancing eHealth literacy can improve physical health and resilience but may unintentionally increase anxiety and depression disparities, necessitating integrated mental health support. Additionally, 4 and 8 disease-pair comparisons showed health disparities only within high and low eHealth literacy groups, respectively, underscoring the importance of developing interventions sensitive to eHealth literacy variations to better manage disease and improve overall health outcomes.

### Disease Disparities in Physical, Mental, and Health Empowerment Outcomes

Previous research indicates that individuals with combined asthma and COPD experience more severe respiratory symptoms, lower quality of life, and more frequent exacerbations than those with a single condition [9,39]. Our study expands upon previous findings, revealing significant disparities not only in health-related quality of life but also in heightened depression and anxiety—likely exacerbated by a heavier symptom burden and frequent exacerbations [40]. Furthermore, the combined COPD-asthma group also exhibits greater nicotine dependence than the asthma group, suggesting that severe symptoms could drive increased nicotine use as a coping mechanism or results from entrenched smoking habits [41,42]. These findings highlight the need for targeted smoking cessation programs and regular nicotine dependence screenings for the combined group. The combined COPD-asthma group also had lower resilience than the COPD group alone, but not the asthma group, possibly due to the challenges of asthma management impairing coping ability [43]. Based on our findings, health care providers should prioritize targeted smoking cessation programs and regular nicotine dependence screenings for individuals with combined COPD-asthma. Additionally, integrating mental health support and resilience-building interventions, such as counseling and support groups, can help manage the increased psychological stress in this group.

### Moderation Effects of eHealth Literacy on Health Disparities

Several health disparities were significant across both high and low eHealth literacy groups, with eHealth literacy either exacerbating or mitigating these differences. The combined COPD-asthma group reported poorer health-related quality of life, sleep quality, depression, and anxiety compared with the COPD and asthma groups. Low eHealth literacy worsened disparities in quality of life and sleep, likely due to reduced capacity for symptom management. Paradoxically, low eHealth literacy was associated with reduced disparities in anxiety and depression among individuals with combined COPD-asthma, perhaps because limited awareness of disease severity confers a “protective” effect on mental health [44]. This phenomenon can be explained by 3 mechanisms: information avoidance, reduced cognitive strain, and built-in stress buffering. Specifically, information avoidance is a coping strategy in which individuals deliberately avoid or minimize exposure to threatening information to protect themselves from distress. High information-seekers often report greater anxiety and even depression, whereas more avoidant individuals may feel less emotional distress in the short term [45]. Second, reduced cognitive strain is explained by cognitive load theory, which suggests our brains have a limited capacity; overwhelming this capacity with excessive information can cause stress, indecision, and mental fatigue [46]. In contrast, those who access less information (whether by choice or due to low e-literacy) may avoid overloading their minds, which can reduce anxiety because they are not constantly processing every possible risk or complication. Third, built-in stress buffering occurs when people with lower eHealth literacy rely on supportive others—such as doctors, nurses, or family members—to interpret health information for them, rather than personally sifting through websites. This reliance can serve as a form of social support and informational filtering, which may reduce anxiety. Additionally, the combined COPD-asthma group also displayed lower resilience than the COPD group across both literacy levels, with disparities being more pronounced in the low eHealth literacy group, possibly due to limited access to supportive digital resources [47]. In practice, interventions should simultaneously enhance eHealth literacy and focus on improving symptom management for quality of life and sleep, while establishing community groups and resilience workshops for practical coping strategies and peer support.

For individuals with low eHealth literacy, user-friendly digital tools and offline resources are crucial to supporting their current eHealth literacy level. Most importantly, although low eHealth literacy may confer certain “protective” effects on mental well-being, it is essential to strike a balance—complete avoidance of information is not ideal for physical health outcomes. The challenge is to provide patients with appropriate, digestible information and strong support so they feel informed but not overwhelmed. This approach harnesses the protective aspects of low eHealth literacy (avoiding unnecessary stress) while ensuring patients have the critical knowledge they need to manage their health. Meanwhile, eHealth literacy improvement programs remain essential, and mental health support for individuals undergoing eHealth literacy enhancement is crucial to avert unintended disparities in mental health outcomes. Integrated eHealth literacy improvement programs that combine digital education, while considering the barriers [48,49], with psychological support can be highly beneficial. Specifically, patients should be encouraged to adopt selective searching, with tiered or “layered” information so that those who feel overwhelmed can stick to the basics while deeper details remain accessible by choice. Additionally, fostering peer-support networks and involving families in health care decision-making can also help filter information [50], reducing anxiety and confusion. Finally, integrating coping strategies—such as CBT-based modules or mindfulness training—can help patients manage distress [51], recognize cognitive distortions, and engage in gradual exposure to potentially unsettling medical information.

Only 4 disease pair comparisons showed significance in health disparities solely in the high eHealth literacy group. Both COPD and the combined COPD-asthma groups exhibited higher nicotine dependence than the asthma group, likely because persons with asthma and high eHealth literacy were more aware of smoking risks and more likely to quit [52], while those in the COPD and combined COPD-asthma groups struggled with entrenched smoking habits despite high literacy. Tailored smoking cessation programs should address these habits through educational campaigns, counseling, nicotine replacement therapies, and motivational interviewing to provide practical quitting support. Additionally, patients with COPD exhibited greater appetite than those in asthma and combined COPD-asthma groups, possibly due to higher adherence to dietary restrictions, such as avoiding allergens and foods that trigger asthma onset, thereby reducing appetite among the latter 2 groups [53]. Enhancing appetite management for those with high eHealth literacy in asthma and combined COPD-asthma groups can be improved with personalized nutrition counseling, allergy-specific eating plans, and regular dietary updates delivered via advanced digital platforms.

Conversely, 8 disease pair comparisons revealed health disparities unique to the low eHealth literacy group. The combined COPD-asthma group exhibited lower stress levels than the COPD and asthma groups, possibly reflecting the “protective” ignorance associated with limited disease awareness common in low eHealth literacy [44], thereby feeling less threatened. However, this group also showed lower self-efficacy, social support, and resilience, likely due to the challenges of managing dual conditions alongside limited access to empowering resources and support networks [47]. Moreover, those with COPD exhibited poorer sleep quality, which is linked to widespread nocturnal symptoms and disturbances affecting over 75% of persons with COPD. These issues are further exacerbated by low eHealth literacy [54], highlighting the importance of interventions aimed at improving literacy to enhance sleep outcomes in the low eHealth literacy group. Persons with COPD also report higher anxiety levels than those with asthma alone, likely exacerbated by the progressive nature of the disease and uncertainties about the future, which are worsened by low eHealth literacy. Enhancing eHealth literacy can reduce health disparities in low literacy groups. Educational programs should address each disease group’s unique needs by incorporating simple digital tools and offline education to support individuals with limited eHealth literacy. However, improving eHealth literacy may negatively impact mental well-being, requiring additional attention. Therefore, tailored interventions should balance the improvement of eHealth literacy with strategies to address mental health challenges, such as integrating mental health support and resilience training alongside digital education.

### Limitations

This study has limitations affecting its interpretation and generalizability. First, the web-based cross-sectional survey may not represent the broader population, potentially under-representing those without internet access and attracting participants with specific health interests or technological skills, which could skew demographic and clinical characteristics. Second, the cross-sectional nature of this study limits causal inference. Future research should adopt a longitudinal approach to assess temporal changes and causal relationships. Third, although self-reported data are inherently susceptible to biases such as inaccuracies and social desirability, the use of validated questionnaires and the exclusion of highly sensitive questions likely mitigated these concerns. Specifically, reliance on self-reported chronic bronchitis can introduce recall bias and potential misclassification, possibly leading to an over- or underestimation of COPD prevalence. Future studies should incorporate corroborative data collection methods and verify diagnoses using objective measures (eg, medical records or spirometry) to further enhance data validity. Fourth, the small sample size for the COPD-asthma group may limit statistical power and the precision of effect estimates. Fifth, the primary focus is on the moderating effect of eHealth literacy, while subgroup analyses based on eHealth literacy levels are secondary and exploratory. Smaller sample sizes within certain disease groups in these subgroups mean that applying multiple comparison corrections would reduce statistical power and increase the risk of type II errors. Thus, *P* values from these subgroup analyses are uncorrected and should be interpreted with caution. Future studies with larger sample sizes are encouraged to validate these findings while implementing stricter type I error controls. Sixth, our study dichotomized eHealth literacy at the scale’s midpoint because no validated cutoff exists for the Chinese eHEALS, acknowledging that this threshold may not be clinically meaningful. Future research should establish standardized, population-specific cutoffs to improve comparability and interpretation. Last, the study’s focus on China may restrict international applicability, as variations in digital access and quality across countries can influence eHealth literacy and patient outcomes. Future studies should consider these global differences in digital access when examining health outcome disparities.

### Conclusions

Our study found that individuals with both COPD and asthma face greater disparities in most outcomes than those with only COPD or asthma. Health professionals should receive specialized training to manage combined COPD-asthma and provide tailored, intensified interventions for this group. Additionally, health disparities vary by eHealth literacy level; therefore, interventions should be customized accordingly. High eHealth literacy interventions include advanced digital tools such as interactive mobile apps, web-based support communities, and personalized health tracking systems. In contrast, low eHealth literacy interventions involve simplified digital resources, offline educational materials, and hands-on support services. Furthermore, eHealth literacy significantly moderates these disparities: lower literacy worsens quality of life, sleep, and resilience but somewhat worsens depression and anxiety. Many specific disparities are unique to the low eHealth literacy group. Boosting eHealth literacy could mitigate most disparities; however, it may also increase mental health issues. Therefore, integrating mental health support into eHealth literacy programs is essential to address these potential side effects.

## Appendix

Multimedia Appendix 1 Outcome variables, scales, and measurement properties.

## Abbreviations

CHERRIES: Checklist for Reporting Results of Internet E-Surveys
COPD: chronic obstructive pulmonary disease
eHEALS: eHealth Literacy Scale
MET: metabolic equivalent
PHQ-9: 9-item Patient Health Questionnaire
