# Supplementary material for: Physical, Mental, and Health Empowerment Disparities Across Chronic Obstructive Pulmonary Disease, Asthma, and Combined Groups and the Moderating Role of eHealth Literacy: Cross-Sectional Study
Source: J Med Internet Res. 2025 May 5;27:e70822. doi: 10.2196/70822 (PMC12089862; doi:10.2196/70822)
Supplement: Multimedia Appendix 1 [file jmir_v27i1e70822_app1.docx]

**Multimedia Appendix 1:** Outcome variables, scales, and measurement properties.

| **Outcomes** | **Scales** | **Measurement properties** |
| --- | --- | --- |
| **Physical and behavioral well-being** | | |
| Health-related quality of life | EQ-5D-5L scale | Cronbach's alpha: 0.81 |
| Nicotine dependence | Chinese version of the 6-item Fagerström Test for Nicotine Dependence (FTND-6) | Cronbach's alpha: 0.65 |
| Physical activity | The International Physical Activity Questionnaire-7 (IPAQ-7) | - |
| Appetite | Chinese version of the 4-item Simplified Nutritional Appetite Questionnaire (SNAQ) | Cronbach's alpha: 0.69 |
| Sleep quality | Chinese version of the brief Pittsburgh Sleep Quality Index (B-PSQI) | Cronbach's alpha: 0.77 |
| **Mental Well-Being** | | |
| Depression | Chinese version of the Patient Health Questionnaire-9 (PHQ-9) | Cronbach's alpha: 0.86 |
| Anxiety | Chinese version of the General Anxiety Disorder-7 (GAD-7) | Cronbach's alpha: 0.89 |
| Perceived stress | Chinese version of the Perceived Stress Scale-4 (PSS-4) | Cronbach's alpha: 0.85 |
| Resilience | Chinese version of the Connor-Davidson Resilience Scale 2-item (CD-RISC-2) | Cronbach's alpha: 0.63 |
| **Health Empowerment** | | |
| eHealth literacy | The 5-item Chinese version of the eHealth Literacy Scale (eHEALS) | Cronbach's alpha: 0.95 |
| Perceived social support | Chinese version of the Perceived Social Support Scale (PSSS) | Cronbach's alpha: 0.89 |
| Self-efficacy | The 3-item new Chinese version of the General Self-Efficacy Short Form | Cronbach's alpha: 0.94 |
